# Alternative Splicing of the Basic Helix–Loop–Helix Transcription Factor Gene *CmbHLH2* Affects Anthocyanin Biosynthesis in Ray Florets of Chrysanthemum (*Chrysanthemum morifolium*)

**DOI:** 10.3389/fpls.2021.669315

**Published:** 2021-06-10

**Authors:** Sun-Hyung Lim, Da-Hye Kim, Jae-A. Jung, Jong-Yeol Lee

**Affiliations:** ^1^Division of Horticultural Biotechnology, School of Biotechnology, Hankyong National University, Anseong, South Korea; ^2^National Academy of Agricultural Science, Rural Development Administration, Jeonju, South Korea; ^3^Floriculture Research Division, National Institute of Horticultural & Herbal Science, Rural Development Administration, Wanju, South Korea

**Keywords:** alternative splicing, anthocyanin, basic helix–loop–helix, chrysanthemum, MBW complex

## Abstract

Chrysanthemum is an important ornamental crop worldwide. Some white-flowered chrysanthemum cultivars produce red ray florets under natural cultivation conditions, but little is known about how this occurs. We compared the expression of anthocyanin biosynthetic and transcription factor genes between white ray florets and those that turned red based on cultivation conditions to comprehend the underlying mechanism. Significant differences in the expression of *CmbHLH2* were detected between the florets of different colors. *CmbHLH2* generated two alternatively spliced transcripts, designated *CmbHLH2^Full^* and *CmbHLH2^Short^*. Compared with *CmbHLH2^Full^*, *CmbHLH2^Short^* encoded a truncated protein with only a partial MYB-interaction region and no other domains normally present in the full-length protein. Unlike the full-length form, the splicing variant protein CmbHLH2^Short^ localized to the cytoplasm and the nucleus and could not interact with CmMYB6. Additionally, CmbHLH2^Short^ failed to activate anthocyanin biosynthetic genes and induce pigment accumulation in transiently transfected tobacco leaves, whereas CmbHLH2^Full^ promoted both processes when simultaneously expressed with CmMYB6. Co-expressing CmbHLH2^Full^ and CmMYB6 also enhanced the promoter activities of *CmCHS* and *CmDFR*. Notably, the Arabidopsis *tt8-1* mutant, which lacks red pigmentation in the leaves and seeds, could be complemented by the heterologous expression of CmbHLH2^Full^, which restored red pigmentation and resulted in red pigmentation in high anthocyanin and proanthocyanidin contents in the leaves and seeds, respectively, whereas expression of CmbHLH2^Short^ did not. Together, these results indicate that CmbHLH2 and CmMYB6 interaction plays a key role in the anthocyanin pigmentation changes of ray florets in chrysanthemum. Our findings highlight alternative splicing as a potential approach to modulate anthocyanin biosynthesis in specific tissues.

## Introduction

Chrysanthemum, one of the most popular ornamental plants worldwide, is an important floriculture crop. Flower color is a key trait for its commercial value, with a differential accumulation of secondary metabolites determining ray floret color. Whereas white ray florets in chrysanthemum accumulate flavone derivatives such as apigenins and luteolins but not anthocyanins, pink and red ray florets accumulate the cyanidin derivatives anthocyanins (Chen et al., 2012; Brugliera et al., 2013; Park et al., 2015).

Anthocyanins play an important role in attracting pollinators and seed dispersers due to their bright colors (Harborne and Williams, 2000; Hoballah et al., 2007). In addition, anthocyanins enhance plant survival under several biotic and abiotic stresses (Chalker-Scott, 1999; Ahmed et al., 2014), which could help plants better adapt to climate change. Anthocyanin accumulation is usually induced under adverse environmental conditions such as high light, nutrient depletion, and low temperature, suggesting that anthocyanins protect cells by scavenging free radicals (Nakabayashi et al., 2014; Schulz et al., 2016). Under natural cultivation conditions, the white ray florets of chrysanthemum flowers often turn red due to anthocyanin accumulation, depending on the genetic background (Xiang et al., 2019).

Extensive studies exploring the molecular mechanism of anthocyanin biosynthesis have led to the isolation and functional verification of regulators that promote anthocyanin biosynthesis in several plant species (Grotewold, 2006; Lin-Wang et al., 2010; Lim and Ha, 2013). A ternary complex composed of MYB–basic helix–loop–helix (bHLH)–WD40 (MBW) family members functions as the major transcriptional regulator of flavonoid biosynthetic genes (Hichri et al., 2011; Xu et al., 2015). In particular, subgroup IIIf bHLH proteins involved in flavonoid biosynthesis share common features, including an MYB-interaction region (MIR) at the N-terminus, a WD40-interacting region in the acidic domain (WD/AD), a bHLH domain, and a C-terminal region, which mediates the formation of homodimers or heterodimers with other bHLH proteins (Heim et al., 2003; Feller et al., 2006). Furthermore, mutations in the coding sequences of bHLH proteins, including PhAN1, IpIVS, and BnTT8, lead to reduced expression of flavonoid biosynthetic genes, thereby resulting in little or no pigment accumulation (Spelt et al., 2002; Park et al., 2007; Chen et al., 2012). These findings suggest that bHLH proteins play crucial roles in anthocyanin biosynthesis by modulating interactions with protein partners such as MYB and/or WD40 proteins.

The MYB and bHLH transcription factors (TFs) CmMYB6 and CmbHLH2 promote anthocyanin biosynthesis in chrysanthemum flowers (Liu et al., 2015; Xiang et al., 2015). In addition, the expression of the recently identified R3 MYB repressor gene *CmMYB#7* is negatively correlated with anthocyanin biosynthesis (Xiang et al., 2019). Thus, CmMYB#7 might inhibit anthocyanin biosynthesis by interacting with CmbHLH2. However, it does not directly regulate the expression of anthocyanin biosynthetic genes, suggesting that competition between CmMYB#7 and CmMYB6 for binding to CmbHLH2 modulates anthocyanin biosynthesis. Therefore, the regulation of anthocyanin biosynthesis appears to be highly dependent on the presence of a partner capable of interacting with CmbHLH2.

Here, we isolated the *CmbHLH2* and *CmMYB6* TF genes and investigated their roles in anthocyanin biosynthesis in the white-flowered chrysanthemum cultivar “OhBlang” (OB), which produces red ray florets at low temperatures. Notably, we observed that the *CmbHLH2* transcript is alternatively spliced. We expressed these transcripts transiently in tobacco leaves and stably in Arabidopsis plants to examine the roles of alternative splicing forms of CmbHLH2 in anthocyanin biosynthesis. Functional characterization of CmbHLH2 variants (CmbHLH2^Full^ and CmbHLH2^Short^) revealed that CmbHLH2^Short^ could not initiate anthocyanin biosynthesis because it cannot interact with CmMYB6. Our identification and analysis of splicing variants of *CmbHLH2* thus increase our understanding of the mechanism underlying anthocyanin pigmentation in the chrysanthemum cultivar OB.

## Materials and Methods

### Plant Materials

Chrysanthemum (*Chrysanthemum morifolium* Ramat.) cultivar “OhBlang (OB)” was used in this study. This cultivar produced either white or red flowers cultivated in a greenhouse from October 2018 to March 2020 at the National Institute of Horticultural and Herbal Science (Wanju, Korea). Chrysanthemums cv. OB that grown in greenhouses at 17–25°C on the timing of flower bud emergence exhibited the fully white-flowered (WO). However, chrysanthemums cv. OB was cultivated below 15°C for 2–3 weeks on the timing of flower bud emergence. It turned pigmentation in the ray florets. We called it turning red OB, TRO. Both white OB (WO) and turning red OB (TRO) flowers were collected at the full-bloom stage. Ray florets were collected from WO and TRO flowers, frozen in liquid nitrogen, and stored at −80°C. Gene expression in the ray florets was analyzed by quantitative PCR (qPCR) and reverse-transcription PCR (RT-PCR).

Transformation experiments were conducted using the *Arabidopsis thaliana transparent testa 8-1* (*tt8-1*) mutant (SALK_030966), which was obtained from the Arabidopsis Biological Resource Center (ABRC). All plants were grown on Murashige and Skoog (MS) medium or soil under long-day conditions (LD, 16-h light/8-h dark, 100 μmol m^−2^ s^−1^) at 22°C. In addition, transient expression experiments were conducted using tobacco (*Nicotiana tabacum*) plants grown in a greenhouse under natural light at 28°C.

### RNA Extraction, cDNA Synthesis, and Genomic DNA Isolation

Total RNA was extracted from ray florets using Fruit-mate for RNA Purification solution (Takara, Otsu, Japan) and Plant RNA Purification Reagent (Invitrogen, Carlsbad, CA, United States) as described previously (Lim et al., 2020) and purified using a FavorPrep™ Plant Total RNA Mini Kit (Favorgen, Changzhi, Taiwan). Total RNA was prepared from tobacco leaves using TRIzol reagent (Invitrogen) and purified using a FavorPrep™ Plant Total RNA Mini Kit (Favorgen), according to the manufacturer’s instructions. DNA contamination was removed by DNase I treatment (Ambion, Thermo Fisher Scientific), and the first-strand cDNA was synthesized from 2 μg of total RNA using amfiRivert cDNA Synthesis Platinum Master Mix (GenDEPOT, Barker, TX, United States).

Genomic DNA was obtained from chrysanthemum leaves using a Plant Mini Kit (Qiagen, Valencia, CA, United States) according to the manufacturer’s instructions.

### Gene Cloning and Sequence Analysis

The full-length open reading frames (ORFs) of *CmbHLH2* and *CmMYB6* were amplified from cDNA and genomic DNA by PCR with PrimeSTAR® HS DNA Polymerase (Takara) using primer sets (*CmbHLH2* and *CmMYB* F/R) listed in Supplementary Table S1. In addition, all PCR fragments were subcloned into the pENTR/D-TOPO vector (Invitrogen) to validate their DNA sequences.

The nucleotide sequences deduced amino acid sequences, and ORFs of *CmbHLH2* and *CmMYB6* were analyzed online.^1^ Structural analysis of the deduced protein was carried out using the ExPASy Molecular Biology Server.^2^ Multiple sequence alignments were generated using the CLUSTALW program.^3^ A phylogenetic tree was constructed using the neighbor-joining method (Saitou and Nei, 1987) with MEGA version 4 software (Kumar et al., 2001).

### qPCR and RT-PCR Analysis

The expression levels of specific genes were quantified by qPCR using AccuPower 2x Greenstar qPCR Master Mix (Bioneer, Daejun, Korea) and the BioRad CFX96 Detection System (BioRad Laboratories, Hercules, CA, United States) according to the manufacturer’s instructions. Gene expression was normalized to *Elongation factor 1α* (*CmEF1α*) and *Glyceraldehyde 3-phosphate dehydrogenase* (*NtGAPDH*) expression for chrysanthemum and tobacco, respectively, as internal references. Three biological replicates were performed for each sample. The primers used for qPCR analysis are listed in Supplementary Table S1.

To validate the alternative splicing of *CmbHLH2*, RT-PCR was performed with specific primers listed in Supplementary Table S1. The resulting DNA fragments were separated on a 1.5% agarose gel with ethidium bromide for visualization.

### Measuring Total Anthocyanin Content

Total anthocyanin content was measured in the samples as described by Lim et al. (2020). Briefly, powdered ray florets from WO and TRO and tobacco leaf samples were incubated in 600 μl extraction buffer (methanol containing 1% HCl) for 6 h at 4°C with moderate shaking. After adding 200 μl water and 200 μl chloroform, the samples were centrifuged at 14,000 *g* for 5 min at 4°C to sediment the plant material. The absorbance of the supernatant was recorded at 530 nm (A_530_) and 657 nm (A_657_) using a microplate reader. Anthocyanin content was determined using the following equation: A_530_ – 0.25 × A_657_. The anthocyanin content in each sample was measured in three independent experiments.

### Subcellular Localization Assay

The subcellular localization of *CmbHLH2^Full^*, *CmbHLH2^Short^*, and *CmMYB6* was analyzed in Arabidopsis protoplasts as described by Yoo et al. (2007). GFP fusion constructs were generated in the p326-sGFP plasmid, which contains the cauliflower mosaic virus 35S (CaMV 35s) promoter. For the C-terminal GFP fusion, the ORFs of *CmbHLH2^Full^*, *CmbHLH2^Short^*, and *CmMYB6* were individually amplified using gene-specific primer sets (p326-*CmbHLH2^Full^*-F/R, p326-*CmbHLH2^Short^*-F/R, and p326-*CmMYB6*-F/R), which introduced an *Xba*I site upstream of the ATG codon, using the InFusion Cloning System (Clontech). The resulting p326-*CmbHLH^Full^*-sGFP, p326-*CmbHLH2^Short^*-sGFP, and p326-*CmMYB*-sGFP plasmids were sequenced to confirm the absence of errors during PCR amplification. The plasmids were introduced into Arabidopsis protoplasts prepared from leaf tissues by polyethylene glycol-mediated transformation. Fusion construct expression was monitored 16–20 h after transformation, and images were captured by fluorescence confocal microscopy (Leica TCS SP8, Leica Microsystems, Germany).

### Transactivation and Yeast Two-Hybrid Assays

To generate the CmbHLH2^Full^, CmbHLH2^Short^, and CmMYB6 BD constructs, sequences encoding complete and partial regions of CmbHLH2^Full^ and CmMYB6 were individually amplified using specific primer sets (Supplementary Table S1). The amplified fragments were cloned into pGBKT7, harboring the GAL4 DNA-binding domain (BD) using an In-Fusion Cloning System (Clontech). The individual BD constructs were transformed into yeast strain AH109 following the manufacturer’s instructions (Takara). The transformed yeast cells were grown on SD medium lacking Trp and replicated on SD medium lacking Trp, His, and Ade containing X-α-gal for color development. After 2 days of incubation in the dark at 30°C, the plates were photographed.

To examine the interactions of CmMYB6 with CmbHLH2^Full^ and CmbHLH2^Short^, three CmMYB6 BD constructs (CmMYB6N1, CmMYB6N2, and CmMYB6N3) were selected, which showed no autoactivation activity in yeast. To validate the protein interactions, complete and partial regions of the CmbHLH2^Full^ coding sequence were individually cloned into pGADT7 harboring the GAL4 AD. The AD and BD constructs were co-transformed into yeast strain MaV203 following the manufacturer’s instructions (Takara). Yeast strains were selected on SD medium lacking Trp and Leu. They were replicated on SD medium lacking Trp, Leu, and His supplemented with 10 mM 3-AT, a competitive inhibitor of the *HIS3* gene product. After 2 days of incubation in the dark at 30°C, the plates were photographed.

### Promoter Activation Assay

The promoter regions of *CmCHS* and *CmDFR* were individually amplified using specific primer sets listed in Supplementary Table S1. The resultant PCR products were purified and cloned into the pENTR/D-TOPO vector (Invitrogen) for sequencing.

The reporter fusion construct was generated by inserting the *CmCHS* and *CmDFR* promoter into the pTr-GUS vector at the 5' end of the *GUS* gene (derived from pBI121) after removing the CaMV 35S promoter region. The constructs were transferred into plant expression vector pBAR as described by Lim et al. (2017) and used as reporter constructs. The ORFs of *CmbHLH2^Full^*, *CmbHLH2^Short^*, and *CmMYB6* were individually subcloned into the pENTR/D-TOPO vector (Invitrogen) and incorporated into Gateway destination vector pB2GW7 (VIB-Ghent University, Ghent, Belgium) using several Gateway cloning steps. The pB2GW7-*CmbHLH^Full^* and pB2GW7-*CmbHLH^Short^* constructs were used as effector constructs. All constructs were transformed into Agrobacterium strain GV3101.

Transient promoter activation assays were performed in *N. tabacum* as previously described (Lim et al., 2017). Briefly, Agrobacteria containing reporter and effector constructs were cultured in LB medium for 2 days at 28°C, pelleted by centrifugation at 3,500 *g* for 5 min at 4°C, resuspended in infiltration buffer (10 mM MgCl_2_ and 100 μM acetosyringone) to an OD_600_ of 0.2 (approximately 10 ml of buffer), and incubated at room temperature without shaking for 2 h. Before infiltration into tobacco leaves, Agrobacteria harboring effector and reporter constructs were mixed at a ratio of 3:1. Then, tobacco leaves were infiltrated with Agrobacteria harboring the genes of interest, and the leaves were harvested to assay GUS activity at 3 days post-infiltration (dpi). Agrobacteria harboring only the GUS reporter construct were used as the control. At least three biological replicates were used for each experiment.

### In Planta Assay of *CmbHLH2* Function

The pB2GW7-*CmbHLH^Full^* and pB2GW7-*CmbHLH^Short^* constructs were individually transferred in Agrobacterium strain GV3101 and transformed into the Arabidopsis *tt8-1* mutant (SALK_030966) using the floral dip method. Transformed Arabidopsis seeds were grown in soil under 16-h light/8-h dark conditions at 20°C. Transgenic Arabidopsis plants were selected by spraying the plants with 0.3% Basta solution. Homozygous T_2_ lines were selected and used for further analysis.

## Results

### Regulatory and Structural Genes in Anthocyanin Biosynthesis Pathways Are Differentially Expressed in Chrysanthemum Ray Florets of Different Colors

**Figure 1:**
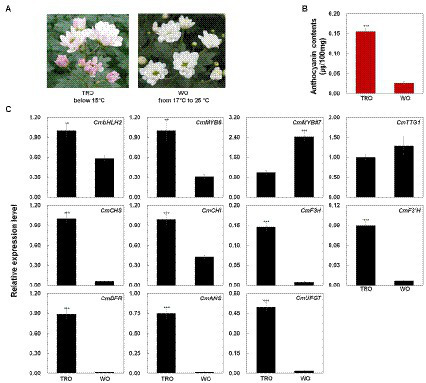
Phenotypes, anthocyanin contents, and anthocyanin biosynthetic gene expression levels in turning red “OhBlang” (TRO) or white “OhBlang” (WO) ray florets. **(A)** Photographs of TRO and WO flowers at the full-bloom stage. **(B)** Anthocyanin level in TRO and WO ray florets. Error bars are the SE of three independent experiments. **(C)** Expression levels of anthocyanin biosynthetic genes and regulatory genes in TRO and WO ray florets. Results represent mean values ± SD from three biological replicates. *CmEF1α* was used as the reference gene. ^**^ and ^***^ indicate values that differ significantly from the control at *p* < 0.01 and *p* < 0.001, respectively, according to Student’s paired *t*-test.

We measured the anthocyanin contents in TRO and WO ray florets (Figures 1A,B). The anthocyanin contents were consistent with the visible appearance of the ray florets. However, only TRO ray florets appeared red. These results indicate that differences in anthocyanin contents are responsible for the color differences in TRO and WO ray florets.

To investigate the mechanism underlying ray floret pigmentation, we examined the transcript levels of genes from TRO and WO at the full-bloom stage (Figure 1C): four regulatory genes, including the R2R3-MYB-type TF gene *CmMYB6*, the bHLH-type TF gene *CmbHLH2*, the WD40 class TF gene *CmTTG1*, and the R3-MYB-type TF gene *CmMYB#7*; and seven structural genes involved in anthocyanin biosynthesis, including early biosynthetic genes (EBGs) encoding chalcone synthase (*CmCHS*), chalcone isomerase (*CmCHI*), flavanone 3-hydroxylase (*CmF3H*), and flavonoid 3' hydroxylase (*CmF3'H*) and late biosynthetic genes (LBGs) encoding dihydroflavonol 4-reductase (*CmDFR*), anthocyanidin synthase (*CmANS*), and UDP glucose: flavonoid-3-O-glucosyltransferase (*CmUFGT*).

*CmBHLH2* and *CmMYB6* transcript levels were higher in TRO vs. WO ray florets, whereas *CmTTG1* transcript levels were similar in TRO and WO. Notably, the transcript level of *CmMYB#7* was negatively correlated with the transcript levels of active regulators, including *CmBHLH2* and *CmMYB6*. In addition, the transcript levels of all anthocyanin biosynthetic genes were significantly associated with the transcript levels of *CmBHLH2* and *CmMYB6*. These results indicate that the high expression levels of *CmBHLH2* and *CmMYB6* were positively correlated with EBG and LBG transcript levels and anthocyanin contents in TRO and WO ray florets.

### Characterization of Regulatory Genes Involved in Anthocyanin Biosynthesis

We generated cDNA sequences of the open reading frames (ORFs) of CmBHLH2 and CmMYB6 to validate the difference in anthocyanin biosynthesis between white and red ray florets primers designed based on previously reported sequences (Liu et al., 2015; Xiang et al., 2015). The *CmbHLH2* cDNA sequences from TRO ray florets shared 100% identity with the previously reported sequence of *CmbHLH2* (GenBank accession number KT724056), a 1,842-bp sequence with an ORF encoding a deduced protein of 613 amino acids, which we refer to hereafter as a CmbHLH2^Full^ (GenBank accession number MW532125). However, the transcript length of *CmbHLH2* from WO ray florets was 1,207 bp with an ORF encoding a deduced protein of 149 amino acids due to the premature stop codon; this predicted protein was designated as CmbHLH2^Short^ (GenBank accession number MW532126), which harbored a large deletion at the C-terminal region (Supplementary Figure S1).

**Figure 2:**
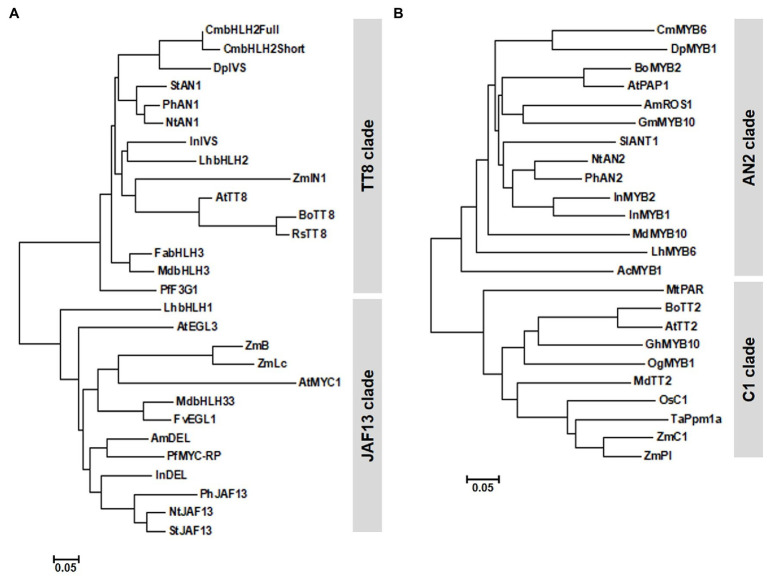
Phylogenetic relationships of anthocyanin biosynthetic regulators in chrysanthemum and other species. **(A)** Phylogenetic tree of CmbHLH2 proteins from WO and TRO and subgroup IIIf bHLH proteins from other plants. **(B)** Phylogenetic tree of CmMYB6 and R2R3 MYB proteins from other plants. The phylogenetic tree was constructed using the neighbor-joining method with MEGA6 software.

Phylogenetic analysis grouped CmbHLH2^Full^ and CmbHLH2^Short^ into the TT8 clade, whose members function in PA and anthocyanin biosynthesis (Figure 2A). Sequence alignment showed that CmbHLH2^Full^ contained well-conserved domains like those of other flavonoid-related bHLH TFs, including an N-terminal MYB-interaction region (MIR) domain, a WD/activation domain (AD), a basic helix–loop–helix (bHLH) domain, and a C-terminal aspartokinase, chorismate mutase, TyrA (ACT)-like domain (Feller et al., 2006; Lim et al., 2017). Furthermore, like other anthocyanin biosynthesis-related bHLH TFs, 19 amino acid residues were well conserved in the bHLH domain. However, CmbHLH2^Short^ contained a partial MIR domain due to alternative splicing, leading to a premature stop codon (Supplementary Figure S1). These results suggest that the generation of an alternative splicing variant of *CmbHLH2* affects anthocyanin biosynthesis in the chrysanthemum cultivar “OhBlang.”

Unlike *CmbHLH2* transcripts, *CmMYB6* transcripts isolated from TRO and WO ray florets shared 100% identity. *CmMYB6* was 765 bp, with an ORF encoding a deduced 254 amino acid. We designated this gene *CmMYB6* (GenBank accession number MW553912). *CmMYB6* encoded a protein with a one-amino acid difference in the R2 domain from the previously reported CmMYB6 protein (GenBank accession number KR002097). In a phylogenetic tree generated from R2R3 MYB proteins from various plant species (Figure 2B), CmMYB6 was grouped into the AN2 clade. Similar to previous findings, sequence alignment revealed that CmMYB6 shared the conserved R2R3 domains in the N-terminal region and the common KPRPR[S/T]F motif in the C-terminal region are conserved in all anthocyanin-promoting MYBs (Supplementary Figure S2).

### Alternative Splicing of *CmbHLH2* in TRO and WO Ray Florets

**Figure 3:**
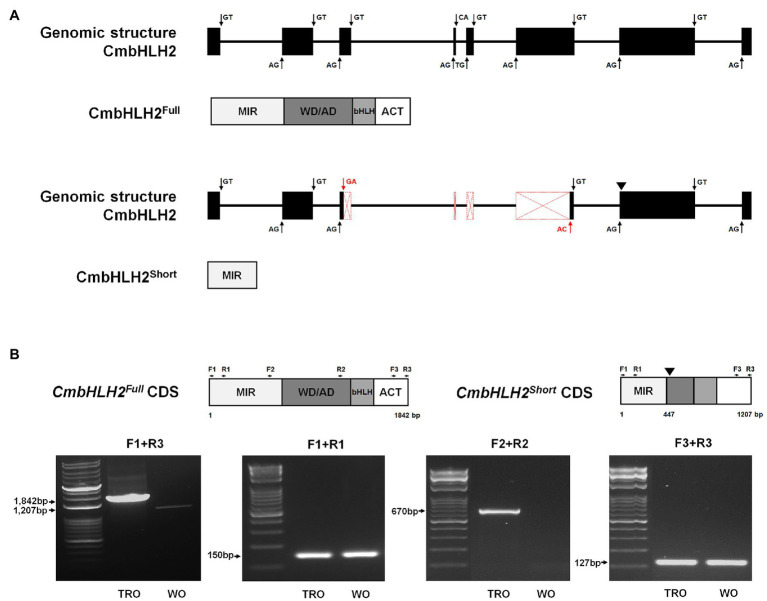
Genomic structure and alternative splicing of *CmbHLH2*. **(A)** Diagram of the genomic structure of *CmbHLH2*. The *CmbHLH2^Full^* and *CmbHLH2^Short^* transcripts were generated by canonical splicing (black arrow) and alternative splicing (red arrow) of *CmbHLH2*. Protein from its mature mRNA is indicated below with functional domains. An inverted triangle indicates the premature stop codon. **(B)** The amplicons’ representative gel image corresponds to *CmbHLH2^Full^* and *CmbHLH2^Short^* transcripts from TRO and WO ray florets. An inverted triangle indicates the premature stop codon. The primers used in this study are shown as arrows and listed in Supplementary Table S1.

To further explore the identity of the splicing variants derived from *CmbHLH2*, we amplified the genomic region of *CmbHLH2*, which was 4,951 bp. Multiple sequence alignment between *CmbHLH2* genomic DNA and the splicing variants revealed that *CmbHLH2^Full^* consisted of 8 exons and 7 introns, but *CmbHLH2^Short^* consisted of only 5 exons and 4 introns (Figure 3A; Supplementary Figure S3). Thus, the deduced CmbHLH2^Full^ polypeptide (predicted from the amplified genomic sequence of mature mRNA) was derived from the primary transcript of *CmbHLH2* containing splicing sites that were indeed recognized by the splicing machinery. By contrast, in *CmbHLH2^Short^* pre-mRNA, an alternative 3' splicing sites in the third exon and 5' splicing sites in the sixth exon) were recognized by the spliceosome producing shorter transcripts. Notably, in these transcripts, the loss of the fourth and fifth exons led to a frameshift, which produced an early stop codon at the beginning of the seventh exon. Thus, the corresponding proteins should show premature truncation, resulting in a much shorter protein than the theoretical one, with the loss of most residues downstream of the MIR domain.

To experimentally determine whether *CmbHLH2* undergoes alternative splicing, we performed RT-PCR using three sets of gene-specific primers targeting different regions of cDNA generated from RNA extracted from TRO and WO ray florets. The gene-specific primer sets (F1 + R1, F2 + R2, and F3 + R3) were designed from the first to second exon, from the fifth to seventh exon, and from the seventh to eighth exon, as shown in Figure 3B. RT-PCR assays with set 1 (F1 + R1) and set 3 (F3 + R3) primers showed no differences between TRO and WO ray florets, indicating that amplicons of the same size were generated (Figure 3B). However, RT-PCR assays with set 2 (F2 + R2) primers amplified products of the expected sizes in TRO ray florets but not in WO ray florets. These findings are consistent with cDNA data showing that the alternative donor site and alternative acceptor site result from the use of cryptic splice sites that might shorten an exon. In addition, these results indicate that *CmbHLH2* generates alternative transcripts in WO ray florets, resulting in the production of a nonfunctional protein with only a partial MIR domain.

### Subcellular Localization Analysis of CmbHLH2 and CmMYB6

**Figure 4:**
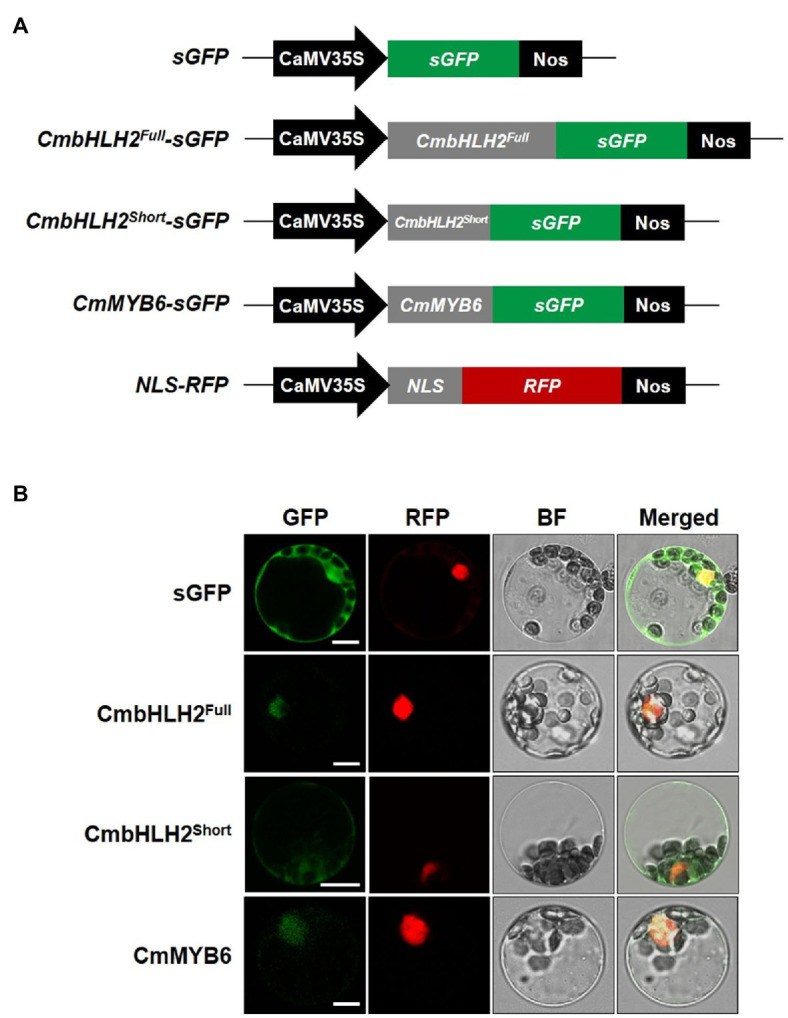
Subcellular localization of CmbHLH2^Full^, CmbHLH2^Short^, and CmMYB6 in Arabidopsis leaf protoplasts. **(A)** Five constructs were used in this experiment: sGFP, soluble GFP; CmbHLH2^Full^::GFP, CmbHLH2^Full^ fused to GFP; CmbHLH2^Short^::GFP, CmbHLH2^Short^ fused to GFP; CmMYB6::GFP, CmMYB6 fused to GFP; and NLS::RFP, nuclear localization signal fused with RFP. **(B)** *In vivo* targeting of CmbHLH2^Full^, CmbHLH2^Short^, and CmMYB6 in Arabidopsis protoplasts. Data are representative of protoplasts expressing fusion proteins at 16 h after transformation. Bar = 10 μm.

Since the subcellular localization of TFs in either the cytoplasm or nucleus regulates their biological activity, we assessed whether the abnormal subcellular distribution of the CmbHLH2 splicing variants could account for differences in anthocyanin biosynthesis. To address this hypothesis, we expressed individual GFP fusions of CmbHLH2^Full^, CmbHLH2^Short^, and CmMYB6 with a nucleus marker construct as a positive control (red fluorescent protein (RFP) fused to the nuclear localization signal (NLS) of the SV40 large T antigen) in Arabidopsis leaf protoplasts and examined their localization by immunofluorescence microscopy (Figure 4A). Protoplasts transfected with CmbHLH2^Full^::GFP and CmMYB6::GFP showed strong GFP fluorescence in the nuclei, but those with CmbHLH2^Short^::GFP showed GFP fluorescence in both the nuclei and cytoplasm (Figure 4B). These results indicate that the alternative splicing form CmbHLH2^Short^ exhibits an abnormal subcellular distribution that could prevent or reduce its interaction with CmMYB6.

### Transcriptional Activation Activity of CmbHLH2^Full^, CmbHLH2^Short^, and CmMYB6

**Figure 5:**
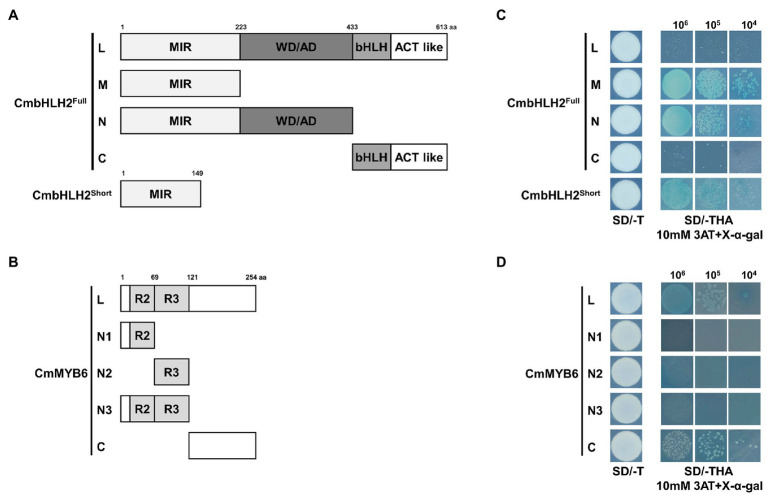
Transactivation assay of CmbHLH2^Full^, CmbHLH2^Short^, and CmMYB6. **(A)** Schematic diagrams of CmbHLH2^Full^ and CmbHLH2^Short^ constructs used in the transcriptional activity assay. **(B)** Schematic diagrams of CmMYB6 constructs used in the transcriptional activity assay. **(C)** Transactivation assay of full-length CmbHLH2^Full^ and relevant truncated proteins and CmbHLH2^Short^ in yeast. **(D)** Transactivation analysis of full-length CmMYB6 and relevant truncated proteins in yeast. Amino acid positions are labeled in the diagrams. SD, minimal medium; AD, activation domain only; BD, binding domain only; 3AT, 3-amino-1,2,4-triazole; SD/−T, minimal medium lacking Trp; SD/−THA + X-α-Gal, minimal medium lacking Trp, His, and Ade but containing 20 mg/ml X-α-Gal; 10^6^, 10^5^, and 10^4^ indicate the number of cells dotted on each plate of the medium.

To determine whether CmbHLH2^Full^, CmbHLH2^Short^, and CmMYB6, have transcriptional activation activity, we generated constructs harboring their gene sequences fused in-frame to the GAL4 DNA-binding domain (GAL4-BD) of the pGBKT7 vector. We assayed their transcriptional activation activity in yeast (Figures 5A,B). We transformed these constructs into yeast strain AH109 and screened the cells on synthetic dropout (SD) medium lacking tryptophan (SD/-T) and triple dropout medium, i.e., SD medium lacking tryptophan, histidine, and adenine (SD/-THA) supplemented with 10 mM 3AT and X-α-Gal.

Among the full and partially truncated clones of CmbHLH2^Full^ and CmbHLH2^Short^, yeast transformants containing CmbHLH2_M, CmbHLH2_N, and CmbHLH2^Short^, which lacked the C-terminal region of CmbHLH2, grew on selection medium and turned blue under the same conditions (Figure 5C). By contrast, yeast colonies (CmbHLH2^Full^ and CmbHLH2_C) containing the C-terminal region of CmbHLH2 failed to grow on the selection medium. These results suggest that the C-terminal region of CmbHLH2^Full^ represses protein autoactivation.

Among yeast transformants containing partial or full-length CmMYB6, yeast transformants containing CmMYB6_N1, CmMYB6_N2, and CmMYB6_N3, which lacked the C-terminal regions of the protein, failed to grow on selection medium and did not turn blue under the same conditions (Figure 5D). By contrast, yeast transformants containing CmMYB6_C, which lacked the N-terminal region of the protein, grew on selection medium, suggesting that the C-terminal region of CmMYB6 is required for its transcriptional activity. Taken together, these results indicate that the full-length CmMYB6 protein possesses strong autoactivation activity and that the C-terminus shows autoactivation activity, but the N-terminus does not (Figure 5D). Therefore, the autoactivation activity of CmMYB6 could be attributed to the C-terminal region.

### Splicing Isoforms of CmbHLH2 Affect Its Interaction with CmMYB6

**Figure 6:**
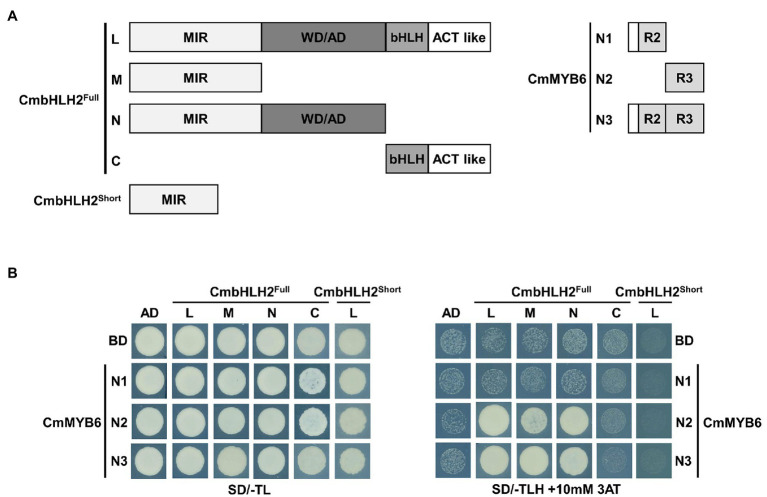
Physical interactions among CmbHLH2^Full^, CmbHLH2^Short^, and CmMYB6. **(A)** Diagram of the constructs used in the Y2H experiment. The amino acid positions of the fragments are numbered. **(B)** Protein–protein interactions among CmbHLH2^Full^, CmbHLH2^Short^, and CmMYB6, as revealed by Y2H analysis. SD/-TL, minimal medium lacking Trp and Leu; SD/-TLH + 3AT, minimal medium lacking Trp, Leu, and His but containing 10-mM 3-amino-1,2,4-triazole (AT). AD and BD indicate the activation domain and binding domain, respectively.

As shown in Figure 1, CmbHLH2 and CmMYB6 together regulate anthocyanin biosynthetic gene expression in chrysanthemum. To examine the interaction between CmbHLH2 and CmMYB6, we constructed bait vectors encoding full-length CmbHLH2^Short^ and complete and partially truncated CmbHLH2^Full^ fragments fused to pGADT7 harboring the GAL4 activation domain (GAL4-AD; Figure 6A) and performed a yeast two-hybrid (Y2H) assay (Figure 6B). As a result, CmbHLH2^Full^_L strongly interacted with CmMYB6_N2 and CmMYB6_N3 but failed to interact with CmMYB6_N1, which harbored the R2 domain. Notably, CmbHLH2^Full^_M and CmbHLH2^Full^_N, which contained the common MIR domain, were able to interact with CmMYB6_N2 and CmMYB6_N3, but not CmMYB6_N1, like the results obtained with CmbHLH2^Full^_L. However, CmbHLH2^Short^, which possessed the partial MIR domain, failed to interact with any proteins with different CmMYB6 regions. These results indicate that the intact MIR domain of CmbHLH2^Full^ and the R3 domain of CmMYB6 are indispensable for interacting with these two proteins.

### CmbHLH2 and CmMYB6 Cooperatively Regulate *CmCHS* and *CmDFR* Promoter Activity

**Figure 7:**
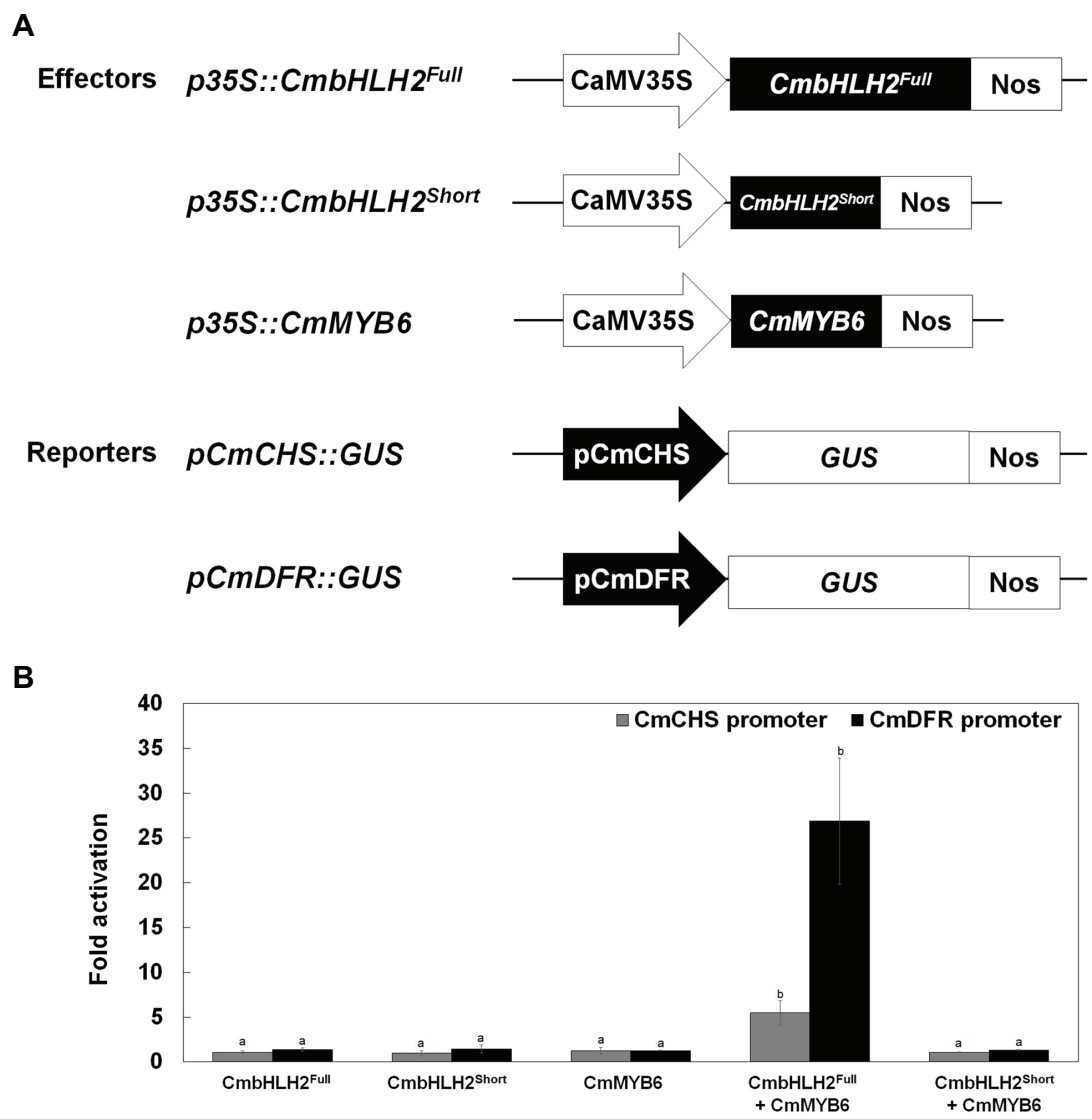
Transcriptional activation assay of the roles of *CmbHLH2^Full^*, *CmbHLH2^Short^*, and *CmMYB6* in activating the *CmCHS* and *CmDFR* promoters. **(A)** Effector and reporter constructs used in the transcriptional activation assay. The effector construct contained *CmMYB6*, *CmbHLH2^Full^*, and *CmbHLH2^Short^* driven by the CaMV 35S promoter. The reporter constructs contained the *GUS* reporter gene driven by the *CmCHS* and *CmDFR* promoters. **(B)** The effects of regulatory proteins CmbHLH2^Full^, CmbHLH2^Short^, and CmMYB6 on *CmCHS* and *CmDFR* promoter activity. Results represent mean values ± SD from three biological replicates. Different letters above the bars indicate significantly different values (*p* < 0.0001) calculated using one-way ANOVA followed by Duncan’s multiple range test.

Various TFs positively and negatively regulate anthocyanin biosynthetic genes. The MYB-recognizing element (MRE) and bHLH-recognizing element (BRE) at the proximal promoter regions of anthocyanin biosynthetic genes are thought to be the targets of MYB and bHLH TFs, respectively (Zhu et al., 2015). To test the activating effects of these TFs on their target genes, we performed a transient transactivation activity assay using *CmCHS* and *CmDFR*, which encode enzymes that catalyze the committed step in flavonoid and anthocyanin biosynthesis, respectively, and contain several MREs and BREs in their promoters (Figure 7A; Supplementary Figure S4). CmbHLH2^Full^ and CmMYB6 were independently infiltrated or co-infiltrated into tobacco leaves along with modified pTr-GUS constructs containing the target *CmCHS* and *CmDFR* promoters driving *GUS* expression (Figure 7B). Neither CmbHLH2^Full^ nor CmMYB6 activated the *CmCHS* or *CmDFR* promoter when expressed separately in tobacco leaves. Furthermore, CmbHLH2^Full^ and CmMYB6 were co-infiltrated into tobacco leaves, GUS activity driven by the *CmCHS* and *CmDFR* promoters increased by approximately 5-fold and 26-fold, respectively. However, in a transient transactivation assay in which CmbHLH2^Short^ and CmMYB6 were expressed separately or together, the *CmCHS* and *CmDFR* promoters were not activated. These results suggest that CmbHLH2^Short^ and CmMYB6 cannot interact with each other, thus failing to activate the *CmCHS* and *CmDFR* promoters. Taken together, these results indicate that a transcriptional complex of CmbHLH2^Full^ and CmMYB6 activates the expression of the *CmCHS* and *CmDFR* promoters more effectively than CmbHLH2 or CmMYB6 alone.

### Simultaneous Expression of *CmbHLH2* and *CmMYB6* Enhances Anthocyanin Accumulation in Tobacco Leaves

**Figure 8:**
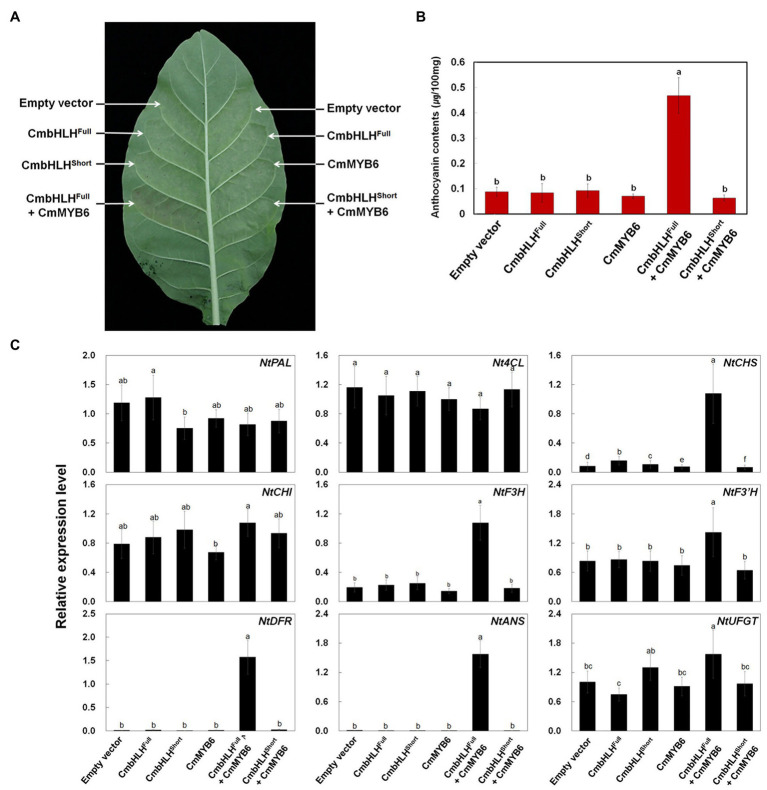
Anthocyanin contents in transiently transformed tobacco leaves infiltrated with Agrobacterium strains carrying *CmMYB6*, *CmbHLH2^Full^*, and *CmbHLH2^Short^*. **(A)** Images of transiently transformed tobacco leaves at 5 days after agroinfiltration. **(B)** Anthocyanin content was extracted from each patch after agroinfiltration. **(C)** Relative expression levels of endogenous anthocyanin biosynthetic genes in tobacco plants, as determined by qPCR analysis. Results represent mean values ± SD from three biological replicates. Different letters above the bars indicate significantly different values (*p* < 0.0001) calculated using one-way ANOVA followed by Duncan’s multiple range test.

To further explore the role of CmbHLH2^Full^, CmbHLH2^Short^, and CmMYB6 in anthocyanin biosynthesis, we conducted a transient expression assay in tobacco leaves (Figure 8A). Similar to the promoter activation assay results, expressing CmbHLH2^Full^, CmbHLH2^Short^, or CmMYB6 alone failed to trigger anthocyanin accumulation in tobacco leaves. However, co-expressing CmbHLH2^Full^ and CmMYB6 resulted in red pigmentation in infiltrated tobacco leaves, whereas co-expressing CmbHLH2^Short^ and CmMYB6 did not. We detected red pigmentation at 5 days post-infiltration (dpi), which gradually increased until 7 dpi. We measured anthocyanin contents in leaf discs collected at 7 dpi. As expected, anthocyanin was barely detectible in leaves infiltrated separately with mock control, CmbHLH2^Full^, CmbHLH2^Short^, and CmMYB6, and in leaves co-infiltrated with CmbHLH2^Short^ and CmMYB6. By contrast, anthocyanin contents in leaves co-expressing CmbHLH2^Full^ and CmMYB6 was a 5-fold increment compared with those in leaves individually expressing CmbHLH2^Full^ or CmMYB6 (Figure 8B).

To further test the roles of CmbHLH2^Full^, CmbHLH2^Short^, and CmMYB6 as regulators of anthocyanin biosynthesis, we analyzed the transcript levels of nine structural genes involved in anthocyanin biosynthesis in infiltrated tobacco leaves by qPCR (Figure 8C). Like anthocyanin contents, the expression levels of anthocyanin biosynthetic genes were similar in leaves infiltrated separately with mock control, CmbHLH2^Full^, CmbHLH2^Short^, or CmMYB6 and in leaves co-infiltrated with CmbHLH2^Short^ and CmMYB6. However, the transcript levels of *NtCHS*, *NtDFR*, and *NtANS* were significantly higher in leaves co-expressing CmbHLH2^Full^ and CmMYB6 vs. the control. In addition, the levels of these gene transcripts were consistent with the anthocyanin levels, and the significant red coloration observed in leaves simultaneously expressing CmbHLH2^Full^ and CmMYB6. Taken together, these results indicate that CmbHLH2^Full^ is essential for controlling anthocyanin biosynthesis together with CmMYB6 in chrysanthemum.

### CmbHLH2 Restores Anthocyanin and Proanthocyanidin Biosynthesis in Arabidopsis *tt8-1* Mutant

**Figure 9:**
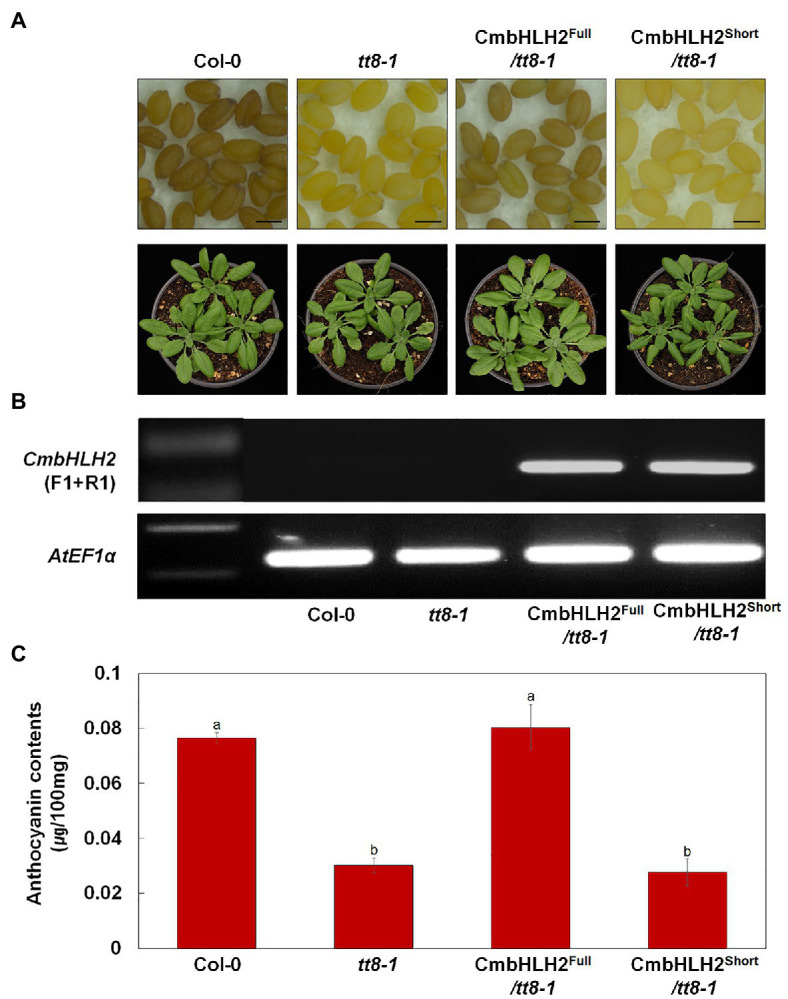
Overexpression of *CmbHLH2^Full^* and *CmbHLH2^Short^* in Arabidopsis *tt8-1* mutants. **(A)** Seeds (top) and 4-week-old seedlings (bottom) of wild-type Col-0, *tt8-1*, and representative T_2_ progeny of homozygous Arabidopsis *tt8-1* lines transformed with *CmbHLH2^Full^* and *CmbHLH2^Short^*, respectively. Scale bar indicates 0.1 mm. **(B)** Expression analysis of *CmbHLH2* in the leaves of wild-type, *tt8-1*, and transgenic plants by RT-PCR. *AtEF1α* was used as the reference gene. **(C)** Anthocyanin content of four-week-old Arabidopsis plants. Results represent mean values ± SD from three biological replicates. Different letters above the bars indicate significantly different values (*p* < 0.0001) calculated using one-way ANOVA followed by Duncan’s multiple range test.

As described above, distinct *CmbHLH2* transcripts were expressed in TRO and WO ray florets. Based on the results of Y2H, promoter activation, and transient expression assays, CmbHLH2^Full^ plays a crucial role in anthocyanin biosynthesis in cooperation with CmMYB6. To investigate the possible roles of CmbHLH2 in flavonoid biosynthesis in planta, we individually expressed *CmbHLH2^Full^* and *CmbHLH2^Short^* under the control of the CaMV 35S promoter in the Arabidopsis mutant *tt8-1*, which lacks anthocyanin in the junction between the stem and rosette leaves and fails to accumulate proanthocyanidin, resulting in yellow seeds (Figure 9A). Following selection with Basta, seeds of T_2_ transgenic lines harboring CmbHLH2^Full^ were brown like wild-type Arabidopsis. By contrast, T_2_ transgenic lines harboring CmbHLH2^Short^ still produced yellow seeds like those of *tt8-1*. We examined the anthocyanin levels in leaves of the transgenic lines harboring CmbHLH2^Full^ or CmbHLH2^Short^ at 4-week-old (Figure 9B). At that stage, we also performed the RT-PCR with primers designed to check for the presence and expression of *CmbHLH2*, determining that the *CmbHLH2* transgene was successfully expressed in all of the transgenic Arabidopsis plants (Figure 9C). Furthermore, anthocyanin levels in leaves from transgenic lines harboring CmbHLH2^Full^ were similar to those of the wild type, whereas anthocyanin levels were not restored to wild-type levels in plants harboring CmbHLH2^Short^. These results demonstrate that the protein encoded by *CmbHLH2^Full^* regulates proanthocyanidin biosynthesis in seeds and anthocyanin biosynthesis in leaves.

## Discussion

The biosynthesis of flavonoids, including anthocyanins and proanthocyanidins, is regulated by ternary MBW (MYB-bHLH-WDR) complexes composed of R2R3-MYB, bHLH, and WDR proteins (Hichri et al., 2011; Xu et al., 2015). Several studies have shown that the differences in anthocyanin levels in the same tissues of certain plant cultivars are mainly attributable to the MYB component of the MBW complex. For examples, in wild-type tomato, alternative splicing of the *SlAN2-like* gene (encoding an active R2R3 MYB) leads to the production of a nonfunctional protein due to the presence of a premature stop codon, resulting in a uniform red color in peels due to the accumulation of only carotenoids in this tissue (Colanero et al., 2020). However, the anthocyanin fruit (*aft*) gene of tomato cultivar Indigo Rose is a functional *SlAN2-like* gene, and mutation of SlMYBATV (a repressive R3 MYB), which acts as a negative repressor by interacting with the MBW complex, results in the accumulation of high levels of anthocyanin in peels (Cao et al., 2017). Similarly, Arabidopsis plants accumulate high levels of anthocyanin in leaves due to the low expression of *AtMYBL2*, the first reported R3 repressor, under both high light and high sucrose levels (Dubos et al., 2008; Matsui et al., 2008). Therefore, anthocyanin production is both positively and negatively regulated by MBW complex formation.

In the current study, we demonstrated that CmMYB6 and CmbHLH2 participate in the regulation of anthocyanin biosynthesis and that the gene products of *CmbHLH2* differ between TRO and WO ray florets. The *CmbHLH2* transcript has changed in splice sites resulting in premature stop codons in the CmbHLH2^Short^ alleles of WO ray florets. *CmbHLH2* transcript levels were correlated with early (EBG) and late (LBG) anthocyanin biosynthetic genes. Additionally, anthocyanin accumulation was only detected following the simultaneous expression of CmbHLH2^Full^ and CmMYB6, but not CmbHLH2^Short^ and CmMYB6 in a transient assay in tobacco leaves. Despite the presence of the *CmMYB6* gene product in TRO and WO, our findings indicate that the gene products of *CmbHLH2* play a crucial role in anthocyanin biosynthesis by functioning as key regulators of ray floret coloration.

Group IIIf bHLH proteins contain several domains, including the MIR, acidic, bHLH, and ACT-like domains, which function in trichome and anthocyanin biosynthesis (Hichri et al., 2011). In *A. thaliana*, the presence of IIIf bHLH proteins harboring only the N-terminal region (MIR and the acidic domains) was sufficient for trichome and non-hair root cell differentiation, but not for anthocyanin biosynthesis (Tominaga-Wada et al., 2012). *Anthocyanin*-less tomato contains a group IIIf bHLH TF with a single nucleotide polymorphism (G to T) that causes G184 to be converted to a stop codon, resulting in the loss of the bHLH domain and the ACT-like domain at the C-terminal region (Qiu et al., 2016). Our results suggest that, like in Arabidopsis and tomato, mutation of group IIIf bHLH proteins modulates anthocyanin biosynthesis in chrysanthemum.

Alternative splicing is when multiple mRNAs from the same gene are generated through the variable selection of splice sites during pre-mRNA splicing. It plays a key regulatory role in modulating gene expression during development and responding to environmental signals (Syed et al., 2012). Under low temperature, it revealed that variation in alternative splicing events might contribute to rapid changes in gene expression and metabolite profile on sugar and antioxidant metabolism (Li et al., 2020). These changes may confer to improve adaptation in plants against environmental stress.

Alternative splicing of structural or TF genes related to anthocyanin biosynthesis affects pigment accumulation in plants. For example, in peach flowers, the *ANS* gene of plants with white flowers exhibits intron retention of a spare 129 bp sequence that is lacking in pink flowers, which leads to the dysfunction of the protein, ultimately blocking the accumulation of colored pigments in white petals (Yin et al., 2019). In tomato, splicing mutations of *R2R3 MYB SlAN2-like*, which control anthocyanin pigmentation, lead to the production of a dysfunctional protein (Colanero et al., 2020). In addition, alternative splicing of the group IIIf bHLH gene *BnTT8* was detected in the yellow seeds of rapeseed (Lin et al., 2020). Interestingly, a recent study reported that the weak allele of *Delila* (*del^23^*), the group IIIf bHLH gene from snapdragon, generated by the alternative splicing due to the insertion in the intron located in the proximal region of the cryptic site resulted in reduced anthocyanin pigmentation in flower (Albert et al., 2021). These findings provide insight into why domesticated crops including tomato, rapeseed, peach, and snapdragon display different pigment accumulation patterns in fruits, seeds, and flowers, respectively. In the current study, *CmbHLH2^Short^*, an alternative splicing variant of *CmbHLH2*, was only detected in WO ray florets and generated a dysfunctional protein inactivation of the *CmCHS* and *CmDFR* promoters and the failure to produce anthocyanin pigments in tobacco leaves and Arabidopsis *tt8-1* plants. Taken together, these results point to an underlying regulatory mechanism for anthocyanin biosynthesis that involves the modification of anthocyanin biosynthetic or TF genes *via* alternative splicing.

This study demonstrated that different *CmbHLH2* transcripts encode truncated proteins lacking the acidic region, bHLH, and ACT domains in WO ray florets. Flavonoid-related bHLH2 TFs contain a MIR motif involved in interactions with the R3 domain of anthocyanin-promoting R2R3 MYB proteins. CmbHLH2^Short^, with a partial MIR motif, was mislocalized to the nucleus and cytoplasm and lost the ability to bind to CmMYB6 (Figures 4, 6). As expected, the simultaneous expression of both CmbHLH2^Short^ and CmMYB6 did not induce anthocyanin accumulation or activate the promoters of *CmCHS* and *CmDFR*. These results suggest that the alternative splicing form of CmbHLH2 prevents the formation of MBW complexes that regulate the expression of anthocyanin biosynthetic genes and thus does not induce anthocyanin production.

Another possible explanation is that the alternative splicing form of *CmbHLH2* might significantly affect anthocyanin levels and anthocyanin biosynthetic gene expression by functioning as a repressor. Indeed, many studies have shown that truncated transcripts can interact with normal transcripts and function as repressors. For example, different alleles of *DvIVS*, a bHLH regulatory gene, affect the regulation of anthocyanin biosynthesis in dahlia (Ohno et al., 2011). The *DvIVS* transcript of the orange ray floret mutant *MJOr* encodes a full-length bHLH regulatory protein, leading to complete orange florets due to anthocyanin accumulation, whereas the *DvIVS* transcript of the yellow ray floret mutant *MJY* encodes a truncated protein lacking the C-terminal region including the bHLH domain due to a Tdv1 insertion into the fourth intron, thereby leading to yellow florets. Morning glory produces different transcripts of *ItIVS*, a bHLH regulatory gene, due to an intragenic tandem duplication, leading to pale flower color (Park et al., 2007). Taken together, these results suggest that truncated bHLH protein not only impedes the conformation of the active MBW complex by directly interfering with the interaction between intact bHLH and MYB, but it also promotes the formation of an inactive MBW complex by replacing normal bHLH protein.

In addition, abnormal *bHLH* transcripts can have altered levels and stability. In dahlia and morning glory, the levels of the aberrant transcripts of *DvIVS* and *ItIVS* (containing premature termination codons) are very low compared with the normal transcripts of wild-type plants, suggesting that the aberrant transcripts might undergo nonsense-mediated decay, leading to degradation of the same mRNAs (Syed et al., 2012; Sibley et al., 2016). Indeed, CmbHLH2^Short^ has retained partial MIR domains but lacks DNA-binding domains and/or transcriptional regulatory domains and cannot interact with the R2R3 MYB CmMYB6. It is possible that the aberrant form competitively interacts with the full-length TF to inhibit its activity.

Overall, we conclude that the TF *CmbHLH2* plays an important role in anthocyanin biosynthesis, depending on the splicing form of its gene. Indeed, alternative splicing of the *CmbHLH2* gene produces at least two transcripts: one encoding a functional TF and an alternative form encoding a dysfunctional TF that negatively regulates the functional form (Seo et al., 2011). Thus, these findings provide insight into a molecular mechanism in which the alternative splicing of *CmbHLH2* modulates the protein interaction network involved in anthocyanin biosynthesis.

## Conclusion

In this study, we demonstrated that anthocyanin biosynthesis in TRO and WO ray florets is determined by the presence of *CmbHLH2*, *CmbHLH2^Full^*, and *CmbHLH2^Short^* transcripts. In the ray florets of chrysanthemum flowers, anthocyanin contents and anthocyanin biosynthetic gene expression strongly depend on the splicing form of *CmbHLH2*. A transient expression assay in tobacco and a promoter activation assay indicated that co-expressing CmbHLH2^Full^ and CmMYB6 activated anthocyanin biosynthesis and the expression of *CmCHS* and *CmDFR* genes expressing CmbHLH2^Short^ and CmMYB6 did not. In addition, a complementation assay of Arabidopsis *tt8-1* revealed that CmbHLH2^Full^ functions in proanthocyanidin and anthocyanin biosynthesis CmbHLH2^Short^ fails to function in these processes. Taken together, these results suggest that the alternative splicing of *CmbHLH2* plays a key role in modulating anthocyanin biosynthesis in the ray florets of chrysanthemum flowers.

## Data Availability Statement

The datasets presented in this study can be found in online repositories. The name of the repository and accession numbers can be found at: National Center for Biotechnology information (NCBI) GenBank, https://www.ncbi.nlm.nih.gov/genbank/, MW532125 and MW532126.

## Author Contributions

S-HL and J-YL conceived, designed the research, and drafted the manuscript. D-HK and J-AJ conducted experiments. All authors contributed to the article and approved the submitted version.

### Conflict of Interest

The authors declare that the research was conducted in the absence of any commercial or financial relationships that could be construed as a potential conflict of interest.

## Footnotes

^1^http://www.ncbi.nlm.nih.gov

^2^http://cn.expasy.org/tools/

^3^https://www.genome.jp/tools-bin/clustalw
