# Supplementary material for: Alternative Splicing of the Basic Helix–Loop–Helix Transcription Factor Gene CmbHLH2 Affects Anthocyanin Biosynthesis in Ray Florets of Chrysanthemum (Chrysanthemum morifolium)
Source: Front Plant Sci. 2021 Jun 10;12:669315. doi: 10.3389/fpls.2021.669315 (PMC8222801; doi:10.3389/fpls.2021.669315)
Supplement: Supplementary file 1 [file Data_Sheet_1.zip › Supplementary Figures.docx]

**Supplementary Figure S1.** Protein sequence alignments of CmbHLH2^Full^, CmbHLH2^Short^ and known anthocyanin-related bHLHs. MIR (MYB-interacting region), acidic WD/AD, bHLH, and ACT-like domains are shaded in different colors. The 19 conserved residues of the bHLH domain are represented using the red boxes. Arrows indicate the HER motif in the bHLH domain.





**Supplementary Figure S2.** Protein sequence alignments of CmMYB6 and known anthocyanin-related R2R3 MYBs. The characteristic R2/R3 domains are shown with grey colors. The anthocyanin-regulating MYB conserved motifs of [A/S/G]NDV and [R/K]Px[P/A/R]xx[F/Y] are represented by red and blue boxes, respectively.


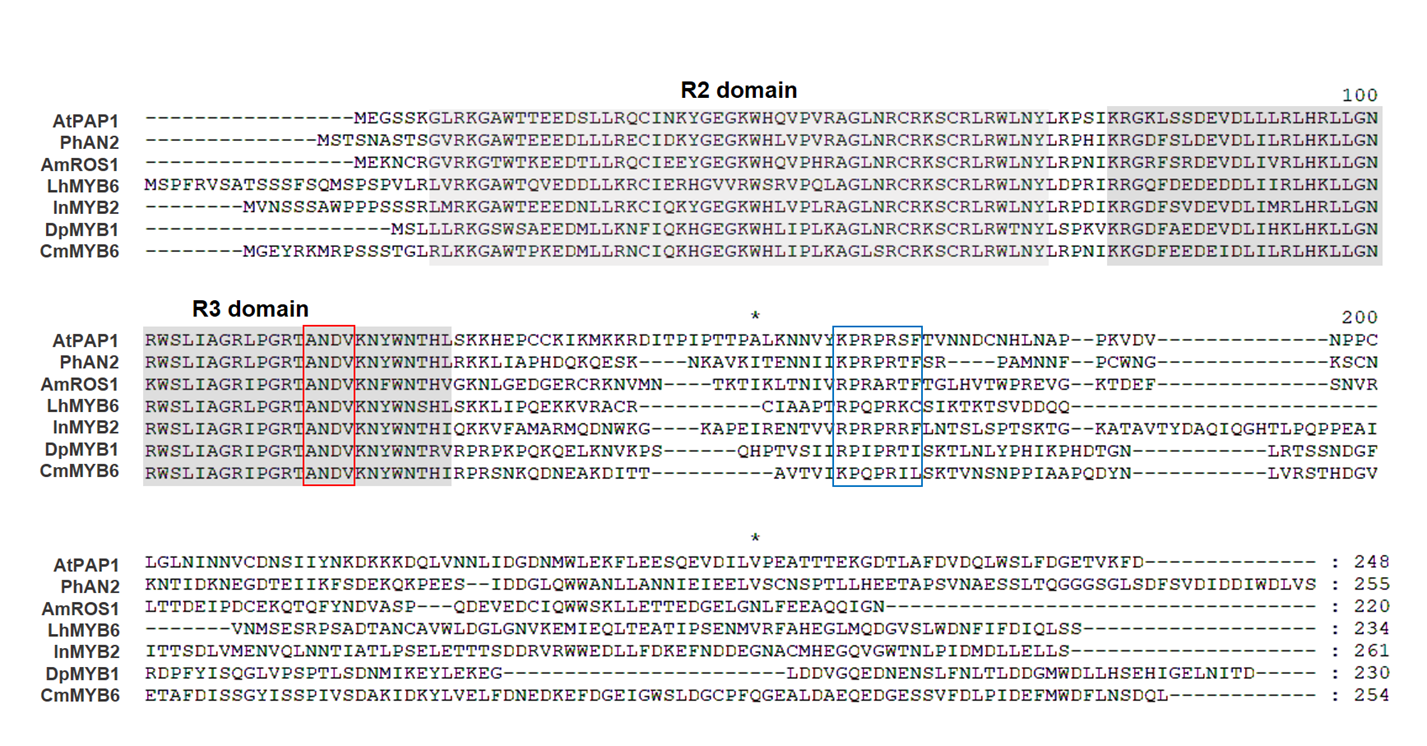


**Supplementary Figure S3.** Alternative splicing of CmbHLH2 gene. Multiple alignment of the nucleotide sequence with genomic DNA of CmbHLH2 and transcripts of CmbHLH2^Full^ and CmbHLH2^Short.^. Exons are shaded in black and grey colors. The roman figures indicate exons. The stop codon is indicated by the red box.


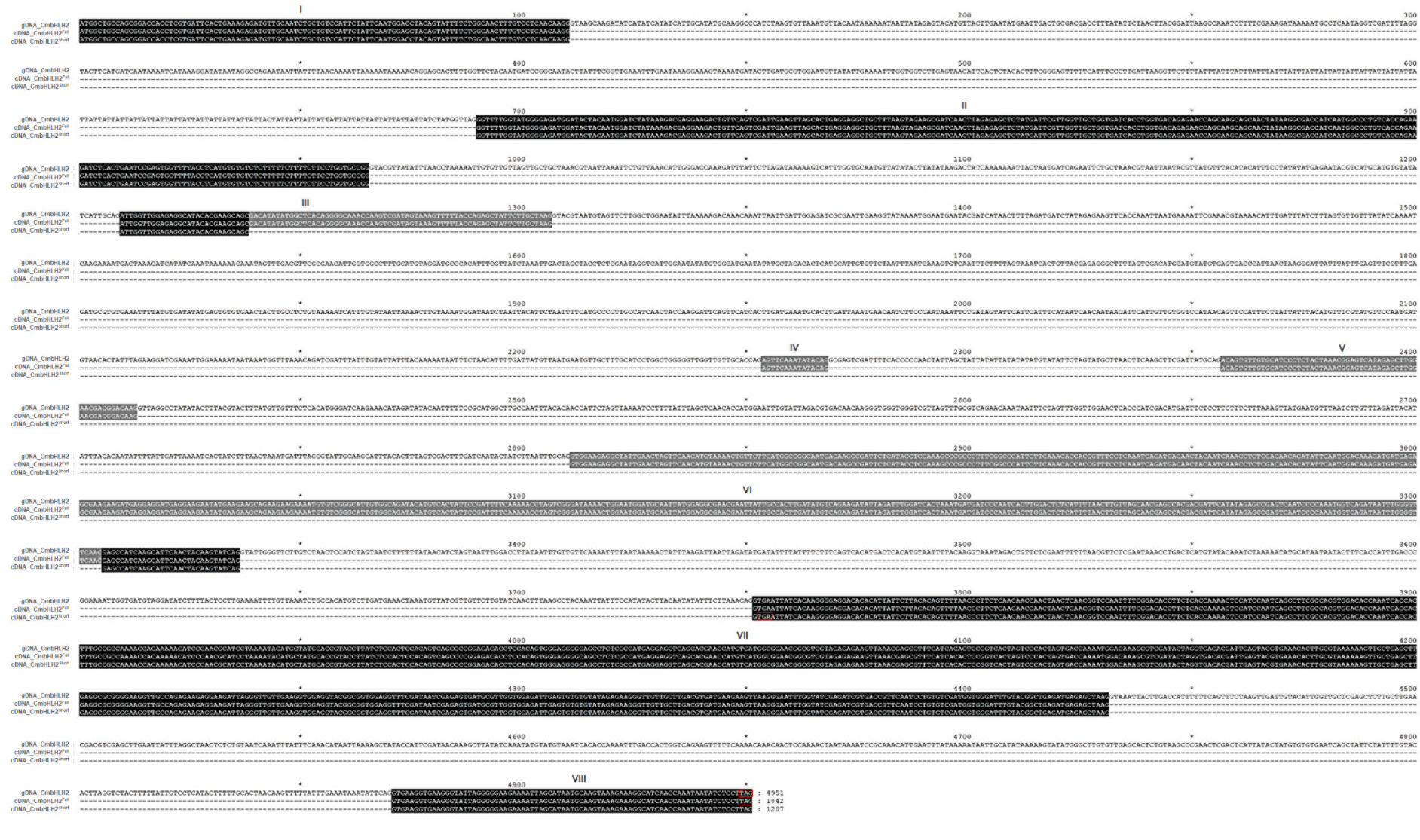


**Supplementary Figure S4**. *CmCHS* and *CmDFR* promoter architectures. (A) Schematic of the *CmCHS* and *CmDFR* promoters showing putative bHLH- and MYB-interacting *cis*-elements involved in anthocyanin biosynthesis. The *cis*-elements are indicated by different symbols. (B) Nucleotide sequence of the *CmCHS* promoter. (C) Nucleotide sequence of the *CmDFR* promoter. BREs and MREs are indicated with red and green boxes, respectively, and the expected TATA box is shown in bold.
