# Supplementary material for: Alternative Splicing of the Basic Helix–Loop–Helix Transcription Factor Gene CmbHLH2 Affects Anthocyanin Biosynthesis in Ray Florets of Chrysanthemum (Chrysanthemum morifolium)
Source: Front Plant Sci. 2021 Jun 10;12:669315. doi: 10.3389/fpls.2021.669315 (PMC8222801; doi:10.3389/fpls.2021.669315)
Supplement: Supplementary file 1 [file Data_Sheet_1.zip › Supplementary Table 1.docx]

**Supplementary Table 1. List of specific primers used in this study.**

| Usage | Primer name | Primer sequence |
| --- | --- | --- |
| Gene expression | qRT-CmbHLH2-F | AGAAGGGTTGTTGCTTGACG |
|  | qRT-CmbHLH2-R | CATCGACACAGGATTGAACG |
|  | qRT-CmMYB6-F | GAATACCAGGAAGAACTGCGAATG |
|  | qRT-CmMYB6-R | GGTTGAGGCTTAATAACTGTGACG |
|  | qRT-CmMYB#7-F | GCCCAGCAGAAGAAGATTTG |
|  | qRT-CmMYB#7-F | CATGTGAATTCCTAAGATTTGG |
|  | qRT-CmTTG1-F | GAGACATCAGGCGAGTGTGA |
|  | qRT-CmTTG1-R | GTTCAATCTCAGCACCAGCA |
|  | qRT-CmCHS-F | CAAGGAGGAGAAGATGAGAG |
|  | qRT-CmCHS-R | CCGAACCCGAATAAAACAC |
|  | qRT-CmCHI-F | GAACCATTTTGATGAGAAAGCA |
|  | qRT-CmCHI-R | TTGCACCCTTAAACTCCTGTT |
|  | qRT-CmF3H-F | AGCGAAGCCCATTGAAAGTA |
|  | qRT-CmF3H-R | TGGCAAGAACACACGCTAAC |
|  | qRT-CmF3'H-F | AGGCGGATTCATCGTTTC |
|  | qRT-CmF3'H-R | ACTCTTTGGGCTTATCAGG |
|  | qRT-CmDFR-F | GCAGCATGGAAAGCAACAAAG |
|  | qRT-CmDFR-R | GGGACTGATAAATGGACCAACAAC |
|  | qRT-CmANS-F | AGGATTAGGACTTGAGGAGGGG |
|  | qRT-CmANS-R | GGTTGAGGGCATTTTGGGTAG |
|  | qRT-CmUFGT-F | TTCCCTTTTGCCTCACACCC |
|  | qRT-CmUFGT-R | TTAAGAACCCTGCGAAACTCCT |
|  | qRT-CmEF1α-F | TTTTGGTATCTGGTCCTGGAG |
|  | qRT-CmEF1α-R | CCATTCAAGCGACAGACTCA |
| Gene cloning | CmbHLH2-F | ATGGCTGCCAGCGGACCACCTCGTGATTC |
|  | CmbHLH2-R | CTAAGGAGATATTATTTGGTTGATGCC |
|  | CmMYB6-F | ATGGGGGAGTACAGAAAAATGAGACCGA |
|  | CmMYB6-R | TCATAGTTGGTCCGAATTTAAAAAGTC |
| Alternative splicing | CmbHLH2-F1 | ATGGCTGCCAGCGGACCACCTCGTGATTC |
|  | CmbHLH2-F2 | ACAAGGTGGAAGAGGCTATTGAAC |
|  | CmbHLH2-F3 | ATCCTGTGTCGATGGTGGGATTTGTACGG |
|  | CmbHLH2-R1 | GATCCATTGTAGTATCCATCTCCCCATACC |
|  | CmbHLH2-R2 | TCCACGTGGCGAAGGCTGATTGGATGGAG |
|  | CmbHLH2-R3 | CTAAGGAGATATTATTTGGTTGATGCC |
| Subcellular localization | p326-CmbHLH2-F | CACGGGGGACTCTAGAATGGCTGCCAGCGGACCA |
|  | p326-CmbHLH2^Full^-R | CCATGGATCCTCTAGAAGGAGATATTATTTGGTT |
|  | p326-CmbHLH2^Short^-R | CCATGGATCCTCTAGACCTGATACTTGTAGTTGAA |
|  | p326-CmMYB6-F | CACGGGGGACTCTAGAATGGGGGAGTACAGAAA |
|  | p326-CmMYB6-R | CCATGGATCCTCTAGATAGTTGGTCCGAATTTAAA |
| Autoactivation and  yeast two hybrid | pGBKT7-CmbHLH2-F | CATGGAGGCCGAATTCATGGCTGCCAGCGGACCA |
|  | pGBKT7-CmbHLH2_C-F | CATGGAGGCCGAATTCATGGTCAGCCCCGGAGACACC |
|  | pGBKT7-CmbHLH2_M-R | GGATCCCCGGGAATTCGGTGTTTGAAGAATGGGCCGA |
|  | pGBKT7-CmbHLH2_N-R | GGATCCCCGGGAATTCGGAGGTGTCTCCGGGGCTGAC |
|  | pGBKT7-CmbHLH2^Full^-R | GGATCCCCGGGAATTCAGGAGATATTATTTGGTT |
|  | pGBKT7-CmbHLH2^Short^-R | GGATCCCCGGGAATTCCCTGATACTTGTAGTTGAA |
|  | pGBKT7-CmMYB6-F | CATGGAGGCCGAATTCATGGGGGAGTACAGAAA |
|  | pGBKT7-CmMYB6_N2-F | CATGGAGGCCGAATTCATAAAGAAAGGAGATTTTG |
|  | pGBKT7-CmMYB6_C-F | CATGGAGGCCGAATTCAAACAAGATAATGAAGC |
|  | pGBKT7-CmMYB6_N1-R | GGATCCCCGGGAATTCATTTGGCCTTAGATAATTT |
|  | pGBKT7-CmMYB6_N2&N3-R | GGATCCCCGGGAATTCGTTGGACCGTGGGCGAATAT |
|  | pGBKT7-CmMYB6-R | GGATCCCCGGGAATTCTAGTTGGTCCGAATTTAA |
|  | pGADT7-CmbHLH2-F | GGAGGCCAGTGAATTCATGGCTGCCAGCGGACCA |
|  | pGADT7-CmbHLH2_C-F | GGAGGCCAGTGAATTCGTCAGCCCCGGAGACACC |
|  | pGADT7-CmbHLH2_M-R | CACCCGGGTGGAATTCGGTGTTTGAAGAATGGGCCGA |
|  | pGADT7-CmbHLH2_N-R | CACCCGGGTGGAATTCGGAGGTGTCTCCGGGGCTGAC |
|  | pGADT7-CmbHLH2^Full^-R | CACCCGGGTGGAATTCAGGAGATATTATTTGGTT |
|  | pGADT7-CmbHLH2^Short^-R | CACCCGGGTGGAATTCCCTGATACTTGTAGTTGAA |

| Transient assay | qRT-NtPAL-F | ATTGAGGTCATCCGTTCTGC |
| --- | --- | --- |
|  | qRT-NtPAL-R | ACCGTGTAACGCCTTGTTTC |
|  | qRT-Nt4CL-F | TCATTGACGAGGATGACGAG |
|  | qRT-Nt4CL-R | TGGGATGGTTGAGAAGAAGG |
|  | qRT-NtCHS-F | TTGTTCGAGCTTGTCTCTGC |
|  | qRT-NtCHS-R | AGCCCAGGAACATCTTTGAG |
|  | qRT-NtCHI-F | GTCAGGCCATTGAAAAGCTC |
|  | qRT-NtCHI-R | CTAATCGTCAATGCCCCAAC |
|  | qRT-NtF3H-F | CAAGGCATGTGTGGATATGG |
|  | qRT-NtF3H-R | TGTGTCGTTTCAGTCCAAGG |
|  | qRT-NtF3'H-F | AGCCATAGTCAAGGAAACC |
|  | qRT-NtF3'H-R | CTCACAACTCTCGGATGC |
|  | qRT-NtDFR-F | AACCAACAGTCAGGGGAATG |
|  | qRT-NtDFR-R | TTGGACATCGACAGTTCCAG |
|  | qRT-NtANS-F | TGGCGTTGAAGCTCATACTG |
|  | qRT-NtANS-R | GGAATTAGGCACACACTTTGC |
|  | qRT-NtUFGT-F | CAATGTTTGGGATGGTGTCA |
|  | qRT-NtUFGT-R | TTCCTCCTCTGCCTCTTTCA |
|  | qRT-NtGAPDH-F | GGTGTCCACAGACTTCGTGG |
|  | qRT-NtGAPDH-R | GACTCCTCACAGCAGCACCA |
| Promoter activation | pCmCHS-F | CAATCATTAATTTGTTTTGGAACATCC |
|  | pCmCHS-R | CGGTGTTTAATATCGGTGAACCAAAATGTA |
|  | pCmDFR-F | AACCCCAACTAGCCATTAGGGGGTGTTTG |
|  | pCmDFR-R | GTTGTTTTAAGCTTGTGGTTTTTGAAGTATTTGTGA |
|  | pTr-pCmCHS-GUS-F | GGCCAGTGCCAAGCTTCAATCATTAATTTGTTTT |
|  | pTr-pCmCHS-GUS-R | GACCACCCGGGGATCCCGGTGTTTAATATCGGTG |
|  | pTr-pCmDFR-GUS-F | GGCCAGTGCCAAGCTTAACCCCAACTAGCCATT |
|  | pTr-pCmDFR-GUS-R | GACCACCCGGGGATCCGTTGTTTTAAGCTTGTGGTT |
|  | pBAR-pCmCHS-F | CCGACGTCGCATGCCTGCAGCAATCATTAATTTGT |
|  | pBAR-pCmDFR-F | CCGACGTCGCATGCCTGCAGAACCCCAACTAGCCA |
|  | pBAR-NOS-R | CTAAGCTTGCATGCCTGCAGGAATTCCCGATCTAGTAACATAG |
| Complemenation test | pUC57mini-CmbHLH2-F | AAAGCAGGCTGAATTCATGGCTGCCAGCGGACCA |
|  | pUC57mini-CmbHLH2^Full^-R | AAAGCTGGGTGGATCCCTAAGGAGATATTATTTGGT |
|  | pUC57mini-CmbHLH2^Short^-R | AAAGCTGGGTGGATCCTCACCTGATACTTGTAGTTGA |
|  | pB2GW7-CmbHLH2-F | AAAAAAGCAGGCTCCATGGCTGCCAGCGGACCA |
|  | pB2GW7-CmbHLH2^Full^-R | GTACAAGAAAGCTGGGTCTAAGGAGATATTATTTGGT |
|  | pB2GW7-CmbHLH2^Short^-R | GTACAAGAAAGCTGGGTTCACCTGATACTTGTAGTTGA |
